# Vection is the main contributor to motion sickness induced by visual yaw rotation: Implications for conflict and eye movement theories

**DOI:** 10.1371/journal.pone.0175305

**Published:** 2017-04-05

**Authors:** Suzanne A. E. Nooij, Paolo Pretto, Daniel Oberfeld, Heiko Hecht, Heinrich H. Bülthoff

**Affiliations:** 1 Department of Human Perception Cognition and Action, Max Planck Institute for Biological Cybernetics, Tübingen, Germany; 2 Department of Experimental Psychology, Johannes Gutenberg-Universität, Mainz, Germany; Tokai University, JAPAN

## Abstract

This study investigated the role of vection (i.e., a visually induced sense of self-motion), optokinetic nystagmus (OKN), and inadvertent head movements in visually induced motion sickness (VIMS), evoked by yaw rotation of the visual surround. These three elements have all been proposed as contributing factors in VIMS, as they can be linked to different motion sickness theories. However, a full understanding of the role of each factor is still lacking because independent manipulation has proven difficult in the past. We adopted an integrative approach to the problem by obtaining measures of potentially relevant parameters in four experimental conditions and subsequently combining them in a linear mixed regression model. To that end, participants were exposed to visual yaw rotation in four separate sessions. Using a full factorial design, the OKN was manipulated by a fixation target (present/absent), and vection strength by introducing a conflict in the motion direction of the central and peripheral field of view (present/absent). In all conditions, head movements were minimized as much as possible. Measured parameters included vection strength, vection variability, OKN slow phase velocity, OKN frequency, the number of inadvertent head movements, and inadvertent head tilt. Results show that VIMS increases with vection strength, but that this relation varies among participants (*R*^2^ = 0.48). Regression parameters for vection variability, head and eye movement parameters were not significant. These results may seem to be in line with the Sensory Conflict theory on motion sickness, but we argue that a more detailed definition of the exact nature of the conflict is required to fully appreciate the relationship between vection and VIMS.

## Introduction

Circular vection is the sensation of self-rotation in stationary observers induced by rotation of the visual surround [1,2]. Traditionally, this apparent self-motion is induced with the participant sitting inside a cylindrical drum, which is painted on the inside with alternating black and white stripes. When the drum starts rotating around the stationary participant, the moving visual pattern evokes an optokinetic nystagmus (OKN), and a compelling sense of self motion in the opposite direction gradually develops over time. A state of “full” vection is reached when the participant perceives the drum to be Earth-stationary, and all visual scene motion is attributed to self-motion.

Prolonged exposure to visual yaw rotation (i.e., 20–30 min) may also induce symptoms of motion sickness (MS, e.g., [3–10]) which, in this case, is referred to as Visually Induced Motion Sickness (VIMS). Symptoms include stomach discomfort, nausea, oculomotor discomfort and dizziness. As will be laid out below, there are different views on why visual yaw rotation provokes such symptoms. Depending on the underlying theory, vection, eye movements (OKN), head movements, and postural instability have been proposed as contributing or causal factors in the genesis of VIMS. In this study we investigate their relative contributions.

The first theory in the context of VIMS is the Sensory Conflict (SC) theory [11], which explains VIMS in terms of conflicting information from the vestibular and visual sensory modality. Specifically, VIMS would occur because of the unfamiliar pattern of stimulation (i.e., visual motion information is not accompanied by vestibular motion information), which is not in agreement with what is expected based on previous experience. Since MS requires either real or apparent self-motion (see p. 1 in [11]) it is argued that vection is a prerequisite for VIMS ([12], see also [13]). The literature on the role of vection in VIMS, however, remains contradictory. Some studies show that participants who suffer from VIMS also experience vection (e.g., [12,14,15]), and that experimental conditions affecting vection also affect VIMS, when assessed on a group level (e.g., [7,9,16–18]). Other studies, however, showed that vection could be manipulated independently of VIMS [19–21], or that VIMS could be elicited in the absence of vection [22]. Based on the notion of sensory conflict, it can be argued that it is not vection *per se* that is important for VIMS, but rather the *changes* in vection intensity. Since the vestibular system only responds to linear or rotational accelerations, these seem to form an essential element in the provocative motion stimulus (see p. 102 in [11]). During circular yaw rotation, such an acceleration occurs at the start of rotation, where the visual velocity step is not accompanied by a vestibular response. When a steady state of vection is reached, (i.e., perceived self-rotation at constant velocity), the vestibular response is no longer expected and thus, the presumed conflict is absent. As this usually occurs within the first minute of exposure, it seems unlikely that this initial conflict is the direct cause of VIMS, because VIMS requires longer exposure to develop. However, when the vection intensity varies during the trial, for instance when the participant drops out of the illusion and it has to build up again, the visual-vestibular conflict would re-appear. As such, changes in vection intensity are believed to contribute to VIMS. This hypothesis has received scant attention in the literature on VIMS (but see [23]).

A second theory that has gained renewed interest in the context of VIMS is the Eye Movement theory [24]. It proposes that reflexive eye movements, such as the OKN during visual yaw rotation, provide eye-muscle afferences that ultimately stimulate the Nervus Vagus [24]. Support for the involvement of the OKN in VIMS is found in studies showing that VIMS severity correlates with OKN frequency [25] and OKN slow phase velocity (OKN SPV, [22]). Furthermore, suppressing the OKN by visual fixation reduces VIMS [9,20]. A complicating factor, however, is that manipulations of the OKN often also affect vection [7,9,17], which makes it hard to disentangle these two factors.

A third theory, proposing yet another causal factor in VIMS, is the Subjective Vertical Mismatch theory [26]. This is actually a refinement of the SC theory proposing that not all sensory conflict are provocative, but only those associated with the sense of verticality. Because pure (visually induced) yaw rotation about an Earth-vertical axis does not affect one's sense of verticality, this can–by itself–not be a provocative stimulus. Instead, as argued by Bles and colleagues, VIMS symptoms may arise because participants make inadvertent head movements while in circular vection. Such head movements cause pseudo-Coriolis effects, which are known to be provocative [27]. Although in many VIMS studies some form of head fixation is used, small head movements may still contribute to VIMS, especially since VIMS symptoms are usually reported verbally at multiple occasions during the trial. Another issue related to this theory is the proper alignment of the drum. Using non-rigid or poorly aligned optokinetic drums may cause the rotation to be perceived about an off-vertical axis, which is provocative and does affect the sense of verticality [28,29].

Finally, it is worth mentioning Postural Instability theory (e.g., [14,30]), which argues that posture control movements are a necessary causal factor in the genesis of MS. A visual stimulus that induces postural instability should produce VIMS. Thus, VIMS should not arise–no matter how salient the visual motion–as long as head and body are immobilized to the extent that postural control remains unchanged.

In the current study, we sought to gain more insight into the role of the above-mentioned factors in VIMS by applying an integrative approach to the problem. First, we wanted to verify whether VIMS indeed occurred after a 20-min exposure to visual yaw rotation when the head was erect and immobilized as much as possible and ample head and body support minimized demands for postural control. Because small head movements or slow position changes might occur even despite proper precautions, head movements were measured and included in further analysis. In the remainder of the paper we will refer to these head movements as *inadvertent*, to stress the fact that they were not intentionally part of the motion sickness inducing stimulus (as e.g. in [15,27]).

Second, we wanted to investigate the role of OKN, vection, and vection variability in VIMS, by systematically manipulating OKN and vection intensity and obtaining continuous measures of all parameters over the course of the trial. It is clear from previous studies on this topic that independent manipulation of vection strength and OKN is difficult to obtain. OKN was suppressed by a visual fixation target, knowing that this would likely affect vection strength [7,9]. It was also expected that the manipulation of vection would affect OKN [7,9]. In previous studies, vection strength was manipulated by field-of-view (FoV) reduction, which indeed decreased but not completely abolished vection [7,9,20]. Here, we followed the approach of Ji and colleagues [22], and introduced an ambiguous vection stimulus consisting of opposite motion directions of the central and the peripheral part of the FoV. When the size of the central part is appropriately tuned, this stimulus has been found to suppress vection completely, while preserving the total amount of visual motion in the entire FoV [2,22]. By applying these manipulations in a full factorial (2x2) within-subjects design, we aimed at obtaining various levels of vection and OKN strength, including a control condition where both were fully suppressed. High levels of VIMS were expected when both OKN and vection were maximal, minimal levels of VIMS in the conditions where both were suppressed, and intermediate levels in the two other conditions. Combining all parameters of vection, OKN and also head movements in a linear mixed regression model allowed us to obtain insight into their relative contributions in VIMS.

## Materials and methods

### Ethics statement

**Fig 1:**
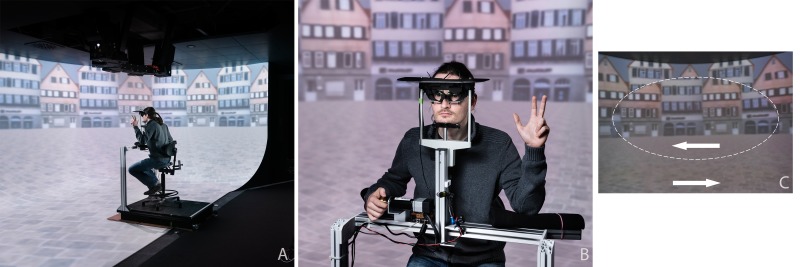
The experimental set-up. (A) Overview of the semi-cylindrical projection screen with floor projection. (B) The participant, here indicating a VIMS score with the left hand, and providing a continuous vection rating with the right. (C) Visual stimulus to suppress vection, where the ellipse-shaped central part of the visual field (dashed line, present in the figure only) moved opposite to the periphery. (A/B: By Berthold Steinhilber / Max-Planck-Institut für biologische Kybernetik)

The experiment was conducted in accordance with the Declaration of Helsinki. All participants gave their written informed consent prior to participation. The experimental protocol and consent forms were approved by the Ethical Board of the Eberhard-Karls-University Tübingen. The person visible in Fig 1A/1B has given written informed consent (as outlined in PLOS consent form) to publish those pictures.

### Participants

Participants were recruited from the Max Planck participant database. All participants had a higher than average susceptibility to motion sickness, as assessed by the Motion Sickness Susceptibility Questionnaire (MSSQ, [31]). That is, their MSSQ-score exceeded the 50^th^ percentile score of the reference population [31]. In total, 18 volunteers were selected (5 males, 13 females), aged between 20 and 48 years (mean age = 28.3 years). They were all free from known vestibular and neurological disorders, and had normal vision, or corrected-to-normal vision while wearing contact lenses (glasses could not be worn in the experimental set-up).

### Experimental set-up

The participant was seated on a height-adjustable chair in the middle of a large panoramic semi-cylindrical projection screen (Fig 1A), which acted as a virtual optokinetic drum. The curved vertical projection area (R = 3.5 m) was seamlessly connected to a projection area on the floor, covering the entire visual FoV (230° horizontal and 125° vertical). Instead of alternating black and white stripes, a pattern of two alternating photorealistic house facades was projected on the inside of the virtual optokinetic dome. Such a naturalistic scene has been shown to induce a more compelling sense of vection than an abstract scene [32]. Similar to several other VIMS studies [7,9,33] the visual scene rotated at a constant speed of 60 deg/s about the Earth-vertical axis.

The participant’s head rested in a head-and-chin rest (Fig 1B), which was carefully positioned in the center of the virtual dome. The upper edge of the projection screen was occluded from the view by a black cap attached to the headrest. The head was further secured by means of an adjustable Velcro strap, which could be opened by the participant in case of emergency. Adjustable back, foot, and armrests provided additional body support. A visual fixation target could be projected on the screen by means of a laser pointer mounted on the head rest.

### Experimental conditions

Each participant was tested in four different experimental conditions, systematically manipulating OKN and vection strength. OKN was manipulated by having a laser fixation target, projected on the screen straight ahead at eye height, presented or not. Vection was manipulated by having the entire visual scene moving coherently in one direction, or by having the central part of the visual scene moving in a direction opposite to that of the peripheral part [2,22]. The central part was ellipse-shaped (Fig 1C), and its size was fine-tuned for each participant to obtain maximum vection reduction (see Procedure and design). In the remainder, the term “ellipse” is used to indicate this directional conflict between the central and peripheral part of the FoV. The manipulations resulted in four conditions: 1) both the ellipse and the fixation target absent, thus maximizing vection and OKN (denoted condition *E*_*off*_*F*_*off*_ with “E” for ellipse and “F” for fixation); 2) both ellipse and fixation target present, thus suppressing vection and OKN (condition *E*_*on*_*F*_*on*_); 3) only the ellipse present, suppressing vection and possibly reducing OKN (condition *E*_*on*_*F*_*off*_); and 4) only the fixation target present, suppressing the OKN while possibly reducing vection (condition *E*_*off*_*F*_*on*_).

### Dependent variables and analysis

In each experimental condition, continuous measures of VIMS, vection, eye movements and head motion were obtained. VIMS was rated using the numerical Fast Motion Sickness scale (FMS, [34]), where a score of zero denotes “I feel fine”, and the maximum score of 20 denotes “I am about to vomit”. Participants gave a non-verbal indication of their score at regular 2-minute intervals (indicated by a beep) during each trial by raising the appropriate number of fingers of their left hand, in multiples of five (Fig 1B). The final number was recorded by the experimenter. For reasons of comparison, VIMS symptoms were also rated before and after the trial using the Simulator Sickness Questionnaire [35]. This questionnaire consists of 16 VIMS symptoms that are rated on 4-point scales. From these ratings a Total Score, as well as three sub-scores related to nausea, disorientation, and oculomotor symptoms were calculated [35].

With the right hand, participants provided a continuous tactile rating of vection strength, using a rotary knob (Sensodrive GmbH, Germany) mounted on the right armrest (Fig 1B). The knob rotated in the participant’s sagittal plane and had a range of 90 deg. The end stops were positioned upward (indicating no vection, scene appears to be moving) and 90deg forward (indicating full vection, scene appears stationary). The knob orientation was sampled at a frequency of 100 Hz, and normalized between 0 (no knob deflection) and 1 (full knob deflection). Comparable methods for continuous tactile estimates of vection strength have been successfully applied by others [32,36]. The overall mean of the continuous vection rating, denoted as vection gain, was taken as a measure of vection strength. Changes in the continuous rating over time, denoted as vection changes, were characterized by two measures. The first was the number of reductions (cf. [23]), defined as the number of times the vection gain decreased by a minimum of 10%. As a more general measure reflecting how often the rating changed, we calculated the standard deviation of the vection gain derivative, which is denoted as *SD(dV)*.

Eye movements of the left eye were recorded by a head mounted, video based eye tracking system (EyeSeeCam^®^, Autronic Medizintechnik, Germany) at a frequency of 220 Hz. Horizontal eye velocity and acceleration were calculated from the recorded horizontal eye position using a numerical three-point differentiation and a Gaussian low-pass filter with a corner frequency of 30 Hz. The OKN fast phases were identified following Behrens and Weiss [37], using an acceleration threshold of 1200 deg/s. These fast phases identified the start and end of each OKN beat, from which the median OKN frequency was determined. After removal of the fast phases from the horizontal eye velocity data, a median filter with a window size of 0.25 s was applied to obtain the slow phase velocity (SPV). Dividing this by the stimulus velocity gave the SPV gain. For the visual fixation conditions a “SPV gain” was calculated using the same algorithm to verify that the nystagmus was suppressed. Nystagmus frequency was set to zero for these conditions.

**Fig 2:**
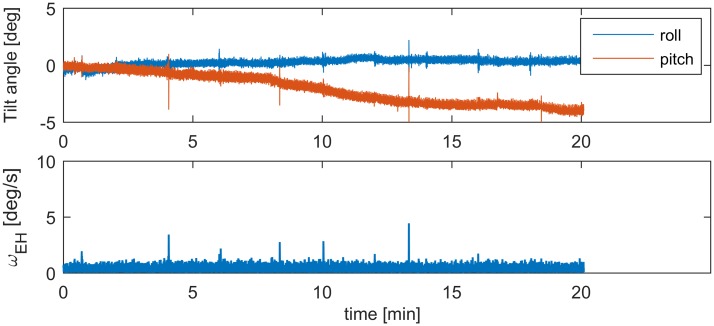
Example of head movement recording, with the head orientation in the upper panel, and Earth-horizontal component of rotational velocity in the lower panel.

Inadvertent head movements were recorded with a small, light-weight motion sensor (3-Space Datalogger, Yost Labs, United States) that was attached to the participant’s head. This sensor provided head angular velocity, linear acceleration, and orientation data, recorded at a frequency of 100 Hz. All data were low-pass filtered with a corner frequency of 10 Hz. A typical example of the head movement data is shown in Fig 2, where it can be seen that there is a gradual change in the absolute head orientation over time (i.e., a semi-static tilt away from the erect, initial position), plus some dynamic head movements that are visible as peaks in both the head orientation and the rotational velocity data. Both the semi-static tilt and the dynamic head movements could be relevant for VIMS, as the first relates to the verticality of the rotation axis, and the second to the occurrence of pseudo-Coriolis effects (see Introduction). For each trial, the magnitude of the semi-static head tilt was identified by fitting a linear trend to the head pitch and roll angles, and then taking the difference between the end and start of the trial. To characterize the dynamic head movements, we calculated the Earth-horizontal component of the angular velocity vector, *ω*_*EH*_ (as only pitch and roll, but not yaw movements cause pseudo-Coriolis effects), and identified the instances where the peak velocity exceeded 3 deg/s. This threshold was chosen to be three times the magnitude of the noise band in *ω*_*EH*_. Apart from the number of head movements, their amplitude and peak velocity was determined. All data processing was performed with Matlab^®^ R2015b (The Mathworks Inc., United States).

### Procedure and design

Using a within-subjects design, participants underwent each condition in a separate 1-hour test session. Sessions were planned at the same time of day with at least 2 days in between. The order of conditions was balanced using a Latin-square design. After explanation of the experimental procedures, the participant was seated in the set-up. Chair height, arm, foot and back rests were adjusted allowing the participant to rest the head in a comfortable position in the head rest. After adjusting the head strap and eye tracker, the participant was familiarized in several 1-minute training trials with the visual stimulus and the experimental procedures. Each trial started with eyes closed, and a beep indicated that the visual motion was rotating at constant velocity and the participant could open the eyes. When watching the visual stimulus, the participant was asked to look approximately straight ahead while allowing the eyes to move, without tracking of visual objects. Furthermore, the participant was asked to keep the head as stationary as possible, to refrain from talking, and to close the eyes and remain stationary until all motion sensations had vanished at the end of each trial (or at trial-abortion). In the first training trial, the stimulus was rotating at a constant speed of 30 deg/s, followed by a trial at 60 deg/s (no ellipse, no fixation). The latter speed was used in all subsequent exposures. Depending on the experimental condition at hand, another training trial was run with the visual target (conditions *E*_*off*_*F*_*on*_ and *E*_*on*_*F*_*on*_) and/or the ellipse (condition *E*_*on*_*F*_*off*_ and *E*_*on*_*F*_*on*_) present. If applicable, the ellipse size was tuned per participant to maximize suppression of vection in a maximum of three 1-minute trials. Default ellipse size was 100 deg horizontally and 35 deg vertically. Its horizontal extent was increased in steps of 20 deg when the direction of perceived self-rotation direction was determined by the peripheral part of the display, and decreased when it was determined by the central part. The aspect ratio of the ellipse remained constant during these changes. Size tuning stopped when no or minimal self-rotation was perceived. Across all participants, final ellipse size ranged between 70 and 170 deg, with 110 deg occurring most often (53.3%). After the training, the participant was taken out of the set-up and allowed to go outside for 10 minutes to have a break. Then the participants gave their baseline VIMS score and filled out the Simulator Sickness Questionnaire (SSQ, [35]). Subsequently, the participant was seated once more in the experimental setup and the eye tracker was calibrated. Then the experimental trial started, which lasted for 20 min or was aborted when a FMS score of 15 had been reached. This cut-off score has prevented frank sickness in the past [34]. Afterwards, the participant stepped out of the setup and filled out a post-trial questionnaire including the SSQ, a post-trial FMS score, and a forced-choice question “Do you feel motion sick after this session (Y/N)?”. The participant was allowed to leave the premises when the FMS score had subsided below 3.

### Statistical analysis

To assess the contribution of eye movements, vection and head movements in VIMS, six relevant parameters for which a link with VIMS has been suggested in the literature (see Introduction) were used as predictors in a linear mixed regression model for VIMS severity. These were OKN SPV gain, OKN frequency, vection gain, vection changes (expressed as *SD(dV)*), the number of inadvertent head movements, and inadvertent head tilt in the roll plane. The latter was preferred above head tilt in the pitch plane, because the vertical structures in the visual stimulus make roll tilt more easily noticeable to the participant than pitch tilt. The number of head movements during the trial was corrected for exposure duration by transforming them into a head movement frequency score. VIMS severity was characterized by the maximum FMS score (occurring usually at the end of the trial), that was also corrected for exposure duration (*FMS*_*norm*_ = *FMS*_*max*_/exposure time). This normalization enabled us to account for differences in symptom severity in case the trial was aborted prematurely. All variables were *z*-transformed. The data were analyzed using a random-effects multiple regression model with random intercept (cf.[38]), taking into account the repeated-measures structure of the data. The variance-covariance matrix was specified as being of type "unstructured"(UN, [39]), and the degrees of freedom were computed according to the method by Kenward and Roger [40], which was demonstrated to be superior to alternative methods [41–43] Because the objective of this analysis was to identify the contribution of each predictor to the VIMS, rating, only data from participants who indeed experienced VIMS in at least one of the four experimental conditions were included in this analysis (*n* = 12). Following recommendations of Belsley et al. [44], influential cases were identified by analyzing the externally studentized residuals and computing the so-called DFFITS index [44]. Observations for which the absolute value of the externally studentized residual exceeded 1.96 or with an absolute DFFITS value exceeding $1.96\;\sqrt{p/N}$ (where *N* is the number of observations and *p* the number of predictors) were defined as outliers and excluded from the data analysis. This occurred for 7 of the 48 included observations. An *R*^2^ statistic for fixed effects [45] was calculated as a measure of the variance accounted for by the multiple regression model, and for calculating partial *R*^2^ for each predictor. In this approach, the full model containing all fixed effects (predictors) is compared to a null model with all fixed effects except the intercept deleted while retaining the same covariance structure. Thus, the *R*^2^ statistic measures how well the variation in the VIMS rating across the four conditions within a given participant is accounted for by a linear combination of the predictors. The analysis was performed in SAS^®^ using the MIXED procedure, with a restricted maximum likelihood (REML) optimization method.

## Results

Data of three of the 18 participants was excluded from further analysis, as one did not meet the MSSQ selection criterion, one reported a fever in one of the test sessions, and one aborted the experiment for non-VIMS reasons.

### Effect of the manipulations on VIMS

**Fig 3:**
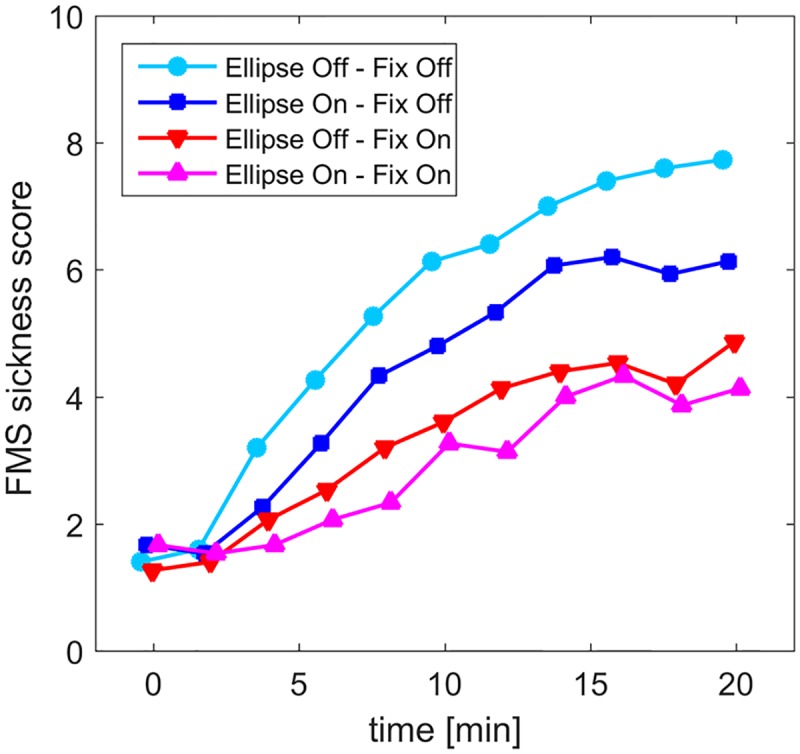
Mean FMS-scores as a function of exposure time for the four conditions. For display purposes, in this figure the final score (FMS 15) was maintained for the remaining time points in case the trial was aborted prematurely.

Fig 3 shows development of VIMS over the course of the trial, whereas further descriptive statistics are provided in Table 1. Exposure to the visual stimulus lead to a gradual raise in FMS scores with time. Paired sample *t*-tests between the maximum FMS scores (*FMS*_*max*_) and the FMS score at the start of the trial showed that this increase was significant in all conditions (*E*_*off*_*F*_*off*_: *t*(14) = 4.7, *p*<0.001;*E*_*on*_*F*_*off*_: *t*(14) = 5.8, *p*<0.001; *E*_*off*_*F*_*on*_: *t*(14) = 4.7, *p*<0.001; *E*_*on*_*F*_*on*_: *t*(14) = 4.7, *p* = 0.004). Over the whole group, the average *FMS*_*max*_ score was highest in the reference condition *E*_*off*_*F*_*off*_ (mean = 8.07 SD = 6.1), where four participants had to abort the trial prematurely. *FMS*_*max*_ scores were lowest in condition *E*_*on*_*F*_*on*_ (mean = 4.6, SD = 4.7), where all participants could finish the entire trial. Intermediate values were found for the two other conditions (Table 1). The *FMS*_*max*_ scores were significantly correlated with the second measure for VIMS severity, the SSQ Total Score (Spearman *ρ*(14) = 0.78, *p*<0.001). Also the correlations with all SSQ subscores were significant (Nausea: *ρ*(14) = 0.89; Oculomotor: *ρ*(14) = 0.71, Disorientation: *ρ*(14) = 0.77, *p*<0.001).

**Table 1:** Stimulus characteristics, average *FMS*_*max*_ scores, average exposure times, and number of drop-outs per condition. Standard deviations are shown in brackets.

Condition	Ellipse	Fixation target	FMS_max_ (-)	Drop-outs (#)	Exposure time (min)
***E*_*off*_*F*_*off*_**	off	off	8.07 (6.1)	4	18.1 (3.9)
***E*_*on*_*F*_*off*_**	on	off	6.80 (4.4)	1	19.6 (1.5)
***E*_*off*_*F*_*on*_**	off	on	5.33 (4.5)	1	19.5 (2.1)
***E*_*on*_*F*_*on*_**	on	on	4.60 (4.7)	0	20 (0)

Although the participants were screened for their susceptibility to motion sickness, we still observed a large intra-individual variability in susceptibility to the visual stimulus, as indicated by the large standard deviation in *FMS*_*max*_ scores (Table 1). Three of the 15 participants were relatively unaffected by the visual stimulation, as they rated themselves as not being motion-sick after *each* of the four sessions. All others (*n* = 12) judged themselves as being sick in at least one session (see Materials and methods).

To statistically determine the effect of our manipulations on VIMS severity, the VIMS scores were first normalized to account for individual differences in exposure time and then submitted to a factorial ANOVA with fixation (2) and ellipse (2) as fixed factors and participant as random factor. This showed a significant main effect of fixation (*F*(1,14) = 9.46, *p* = 0.008), indicating that VIMS was lower when participants fixated the visual target. There also seemed to be a tendency of the ellipse to lower the VIMS scores, but this effect did not reach the 5% significance level (*F*(1,14) = 4.18, *p* = 0.06). There was no interaction between ellipse and fixation (*F*(1,14) = 0.882, *p* = 0.36).

### Effect of the manipulations on the other dependent variables

**Fig 4:**
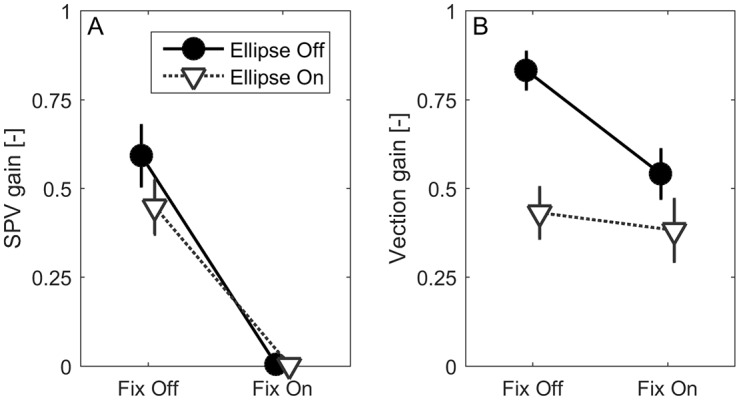
Mean OKN SPV gain (A) and vection gain (B) per condition Error bars indicate standard error of mean.

The main effect of visual fixation on VIMS seems to suggest that OKN, and not vection, plays a major role in VIMS. This, however, would only be an appropriate conclusion if vection was not affected by the fixation target, and if the OKN was not affected not by the ellipse manipulation. Otherwise regression analysis would be the more appropriate to relate these variables to VIMS. We see in Fig 4A that the fixation target indeed suppressed the OKN, as illustrated by the near-zero values for the SPV gain (compare Fix off vs. Fix on). Accordingly, when the SPV-gain values were submitted to a factorial ANOVA with fixation and ellipse as fixed factors and participant as random factor, the main effect of fixation was highly significant (*F*(1,14) = 59.5, *p*<0.001). The main effect of ellipse on SPV-gain (*F*(1,14) = 1.7, *p* = 0.21), and the interaction between fixation and ellipse (*F*(1,13)1.45, *p* = 0.25) were non-significant, with the average gain equal to 0.59 (SD = 0.32) in condition *E*_*off*_*F*_*off*_ and 0.45 (SD = 0.30) in condition *E*_*on*_*F*_*off*_. The ellipse also did not affect the OKN frequency, where a mean value of 4.8 Hz (SD = 1.6) was observed in condition *E*_*off*_*F*_*off*_ vs. 4.5 (SD = 1.5) in condition *E*_*on*_*F*_*off*_ (note that this is not shown in Fig 4, but individual data for this and the other five selected variables is plotted as a function of *FMS*_*norm*_ in the next section).

**Fig 5:**
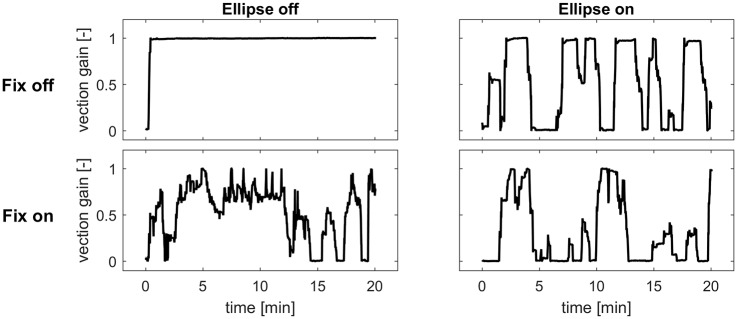
Example of the continuous vection rating for the four conditions (same participant).

Fig 4B shows that the vection gain, on the other hand, is affected by both manipulations, as shown by a significant main effect for fixation (*F*(1,14) = 9.53, *p* = 0.008) and ellipse (*F*(1,14) = 20.40, *p*<0.001). Also the interaction effect was significant (*F*(1,14) = 7.46, *p* = 0.016). Post hoc tests showed that vection gain was highest in the reference condition (*E*_*off*_*F*_*off*_, mean = 0.83, SD = 0.20), and significantly reduced in all other experimental conditions (*E*_*on*_*F*_*off*_: mean = 0.43, SD = 0.28; *E*_*off*_*F*_*on*_: mean = 0.54 SD = 0.27; *E*_*on*_*F*_*on*_: mean = 0.38, SD = 0.34). These latter three conditions did not significantly differ among each other. Complete suppression of vection was not obtained. The relatively large standard deviation in vection gain illustrates that the effectiveness of the ellipse in suppressing vection varied widely among participants. An example of the continuous vection rating for the four conditions is depicted in Fig 5. As also visible in this figure, participants experienced changes in vection strength during the course of the trial, although no differences between the conditions were found. Overall, the average number of vection reductions (see Materials and methods) was 15, ranging from 0 to 60. This measure was correlated with the second measure of vection changes, *SD(dV)* (Spearman *ρ* = 0.75, *p*<0.001). Because we take the latter to be a more general measure of vection gain change during the trial, this parameter was used in the regression analysis described in the next section.

During most experimental trials, inadvertent head movements or inadvertent head tilt was observed, although their amount and amplitude were minimal. The total overall within-trial variation in head orientation (i.e., difference between maximum and minimum value) ranged from 0.9 to 9.0 deg (mean = 3.5; SD = 2.1) for pitch and from 0.7 to 8.8 deg (mean = 2.4; SD = 1.4) for roll. The slow gradual head tilt over the course of the trial was 2.3 deg (SD = 2.1) for pitch and 1.1 deg (SD = 1.0) for roll. Faster head movements (exceeding the 3 deg/s threshold) occurred on average 2.6 times per trial (SD = 4.7), which equals to a frequency of 0.15 head movements per minute (SD = 0.28) after accounting for the individual survival time. Overall, these movements had low peak velocity (mean = 4.5 deg/s, SD = 3.0 deg/s) and low amplitude (mean = 1.5 deg, SD = 1.4 deg). No differences among experimental conditions were observed.

### Relationship between vection, OKN, head movements and VIMS

**Fig 6:**
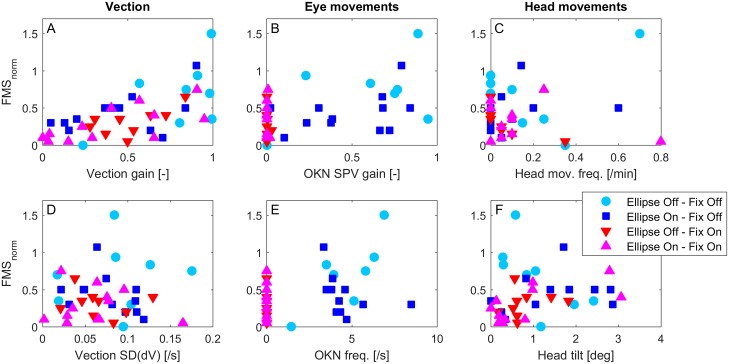
Individual data included in the regression analysis. Each panel relates one of the six predictors to *FMS*_*norm*_. Data is labeled per experimental condition.

To assess whether vection, OKN and/or head movements contributed to VIMS during visual yaw rotation, the six selected parameters with a possible link to VIMS were used as predictors in a linear mixed model with a random intercept for each participant (random intercept model, see Methods). The relationship between these predictors and *FMS*_*norm*_ is shown in Fig 6. With *FMS*_*max*_ = 15 as the stop criterion, and a maximum trial duration of 20 min, *FMS*_*norm*_ > 0.75 indicates a premature trial abortion (when *FMS*_*norm*_ < 0.75, one can easily calculate *FMS*_*max*_ = 20 · *FMS*_*norm*_).

Of all six parameters, vection gain appears to have the most prominent relationship with *FMS*_*norm*_, and indeed, as shown in Table 2, this is the only parameter that has a significant effect on VIMS. It accounts for 39% of the variance [45]. In addition, OKN SPV and head movement frequency account for a small portion of the variance (9%), but their respective regression parameters are not significant. This is illustrated in Fig 6B/6C, showing that high values of *FMS*_*norm*_ are observed at low values of these predictors. This contrasts with the data for vection gain, where high values of *FMS*_*norm*_ are only found at high values of vection.

**Table 2:** Estimates for the fixed effects (regression coefficients) in a random intercept model and in a model also incorporating a random slope for vection gain. All variables were *z*-transformed. *BIC* = Bayesian Information Criterion.

	Random Intercept model				Random slope and intercept model			
	*Estimate*	*SE*	*p*	*R*^*2*^_*partial*_	*Estimate*	*SE*	*p*	*R*^*2*^_*partial*_
Intercept	0.056	0.164	0.741	-	0.021	0.176	0.907	-
Vection gain	**0.410**	**0.096**	**<0.001**	**0.385**	**0.377**	**0.119**	**0.009**	**0.478**
Vection *SD(dV)*	0.067	0.078	0.399	0.028	-0.045	0.080	0.579	0.016
OKN SPV	0.238	0.149	0.122	0.086	0.265	0.144	0.077	0.121
OKN freq.	0.023	0.114	0.840	0.002	0.079	0.098	0.431	0.030
Head tilt	0.022	0.111	0.843	0.001	-0.042	0.099	0.677	0.008
Head mov. freq.	0.213	0.126	0.103	0.087	0.132	0.103	0.213	0.068
*Total R*^*2*^	0.757				0.686			
*BIC*	66.1				63.4			

The model can be further improved by incorporating random slopes for the predictors, to account for individual differences in their relationship with *FMS*_*norm*_. Because of the limited size of our dataset, we chose to include a random slope for the significant parameters only, which in this case is vection gain. The results for this random intercept and slope model are also shown in Table 2. The parameters changed slightly, but the partial *R*^2^ for vection gain increased to 48%, and the partial *R*^2^ of SPV gain to 12%. The partial *R*^2^ of head movement frequency decreased to 7%. The change in the Bayesian Information Criterion (BIC, where smaller is better) shows that, despite the increased number of free parameters in the random slope model, there is positive evidence that the latter is the better model of the two [46].

Because all variables were *z*-transformed before entering the model, it is not immediately intuitive what a change in one of the predictors means in terms of the final parameter of interest, *FMS*_*max*_. We can better appreciate the effect of vection gain on VIMS by applying the model to the data and then transforming the predicted value for the criterion (i.e., the *z*-transformed *FMS*_*norm*_) back into a value of *FMS*_*max*_. When all predictors are set to 0 (i.e., having a *z*-transformed value of $-\bar{x}/\sigma$), the predicted *FMS*_*max*_ score is 1.6 for the random slope model. If we then change the value for the vection gain from 0 to 1 (full vection), the model predicts a value of 11 for *FMS*_*max*_.

It is important to realize that the random-effects regression model measures "correlations within participants” [47]. The positive regression coefficient for vection gain shows that the experimental conditions where the participant produced a higher rating of vection also resulted in a higher VIMS rating. It is, however, not necessarily the case that this relationship also holds *between* participants, that is, that participants with higher vection ratings also have higher VIMS scores than participants with lower vection ratings. Thus, as a complementary to the within-participants analysis presented above, one can compute "correlations between participants" [48]. To that end, the scores on the criterion variable (VIMS) and the scores on the six predictor variables were averaged across the four experimental conditions for each participant. Next, a multiple regression of the VIMS rating on the six predictors showed whether, independent of the experimental condition, high scores in the predictor variables were associated with high scores in the criterion variable. As depicted in Table 3, this regression model explained 72% of the variance in the averaged VIMS rating and indicated a positive association between five of the six predictors and VIMS. However, none of the regression coefficients was significantly different from 0 at an α-level of .05. This indicates that the significant relationship between vection gain and VIMS that was found *within* participants does not hold when assessed *between* participants.

**Table 3:** Estimates for the “between participants” regression model. All variables were *z*-transformed.

	*Estimate*	*SE*	*p*	*R*^*2*^_*partial*_
Intercept	>0.001	0.226	1.000	-
Vection gain	0.412	0.299	0.227	0.275
Vection *SD(dV)*	-0.003	0.340	0.994	0.000
OKN SPV	0.604	0.298	0.098	0.452
OKN freq.	0.414	0.264	0.178	0.330
Head tilt	0.219	0.332	0.539	0.080
Head mov. freq.	0.649	0.282	0.069	0.516
*Total R*^*2*^	0.722			

## Discussion

In the current study, we have investigated the role of vection, eye movements, and inadvertent head movements in VIMS (Visually Induced Motion Sickness) induced by visual yaw rotation. Each of these parameters has been linked to a different motion sickness theory, and the specific stimulus of yaw rotation around a vertical axis was chosen because these theories make different predictions for this case. The Subjective Vertical (SV) theory [26] argues that this stimulus in itself cannot evoke motion sickness and suggests that inadvertent head movements or head tilt might cause VIMS symptoms. The Postural Instability (PI) theory [30], also predicts that such a gravity-neutral stimulus is not provocative, as long as postural responses are not elicited. The Sensory Conflict (SC) theory [11], on the other hand predicts that VIMS would occur and links it to vection, whereas the Eye Movement (EM) theory links VIMS to optokinetic nystagmus (OKN, [24]). The manipulations applied in this study (visual fixation target and conflict in the movement direction of the central and peripheral field of view) were designed to affect OKN and vection strength respectively, whereas head movements and the demands on postural control were minimized as much as possible. However, based on previous research (e.g., [7,9,25]) we anticipated that independent manipulation of vection and OKN would be difficult to obtain. This was indeed found in our data, as vection strength was affected by both manipulations. Thus, instead of focusing on the differences between the four experimental conditions, we assessed the contribution of different parameters for which a link with VIMS could be expected using a linear mixed regression model.

### Vection gain is the main contributor to VIMS

The most important outcome of this analysis is that vection gain is the main contributor to VIMS during Earth-vertical visual yaw rotation. Other parameters (vection changes, OKN slow phase velocity, OKN frequency, frequency of inadvertent head movements and the magnitude of inadvertent head tilt) had non-significant contributions to VIMS. The observed relationship between VIMS and vection gain is in accordance with other studies showing that participants who experience VIMS also experience vection (e.g., [12,14,15]). However, to our knowledge we are the first to show that this relationship holds within participants and not necessarily between participants. That is, for a given participant, an experimental condition eliciting a higher vection gain will also lead to a higher VIMS rating, but participants may differ in the VIMS rating that is related to a certain level of vection. This is explained by the individual differences in VIMS susceptibility, as reflected by the large spread in VIMS ratings in each condition. This large inter-individual difference in VIMS susceptibility might also explain why some studies fail to find a significant correlation between the two variables (e.g., [15,21]). A possible relationship between VIMS and vection may also remain hidden if the rating scales for both vection and VIMS are too coarse to identify differences between experimental conditions, or if the study population includes participants who are not susceptible to VIMS at all. In the current study, we tried to overcome the first issue by using a continuous measure for vection, and a validated fine-grained 20-point rating scale for VIMS (as opposed to 7- or 10-point scales without validation records). The second issue was addressed by screening the participants beforehand. Despite this screening, three of the 15 participants did not suffer from VIMS in the current study. As the screening questionnaire (MSSQ, [31]) only addressed experience with motion sickness provoked by physical stimuli, these results suggest that a VIMS-specific screening should be implemented in future studies.

A last note on vection concerns an apparent paradox about the effect of OKN suppression on vection intensity. Defining vection intensity as a gain ranging from “no vection” to “full vection”, where the latter meant that the visual surround appeared stationary, we found that OKN suppression lead to a lower vection gain (see also [7,9]). This is different from studies that use magnitude estimates of self-motion velocity or angular displacement, and report a higher perceived velocity and/or displacement in this case (e.g., [49]). This increase in vection velocity can be explained by the increased retinal slip velocity during OKN suppression. Thus, although the scene appeared less stationary with visual fixation, the perceived vection velocity can still be higher with visual fixation. The observed relationship between vection gain and VIMS found in the current study suggests that it is not so much the self-motion speed that is important in VIMS, but rather the relationship between perceived self-motion and scene stationarity.

### Contribution of the other parameters

In theory, VIMS could be related not to vection gain per se, but rather to changes in vection gain during the trial. Such changes would re-induce a visual-vestibular conflict and thereby enhance VIMS. For the case of linear vection, some supporting evidence for this hypothesis was found by Bonato and colleagues [23]. The current study is the first to address this issue for circular vection. The data, however, do not support this hypothesis, as changes in vection varied independently of VIMS ratings. Because the experimental conditions were not especially designed to manipulate changes in vection strength during the trial, further research is needed to substantiate these findings. For example, this can be done by varying the rotational velocity of the visual stimulus during the trial, which, indeed, has been found to enhance VIMS [50]. Although we think that such a manipulation can provide further insight into the role of vection changes in VIMS, our finding is robust. Participants who experienced a very constant level of vection still reached high VIMS scores. It is thus rather unlikely that vection changes contributed to VIMS in the current study.

At first glance it might seem surprising that the two OKN parameters, which had been linked to VIMS in previous research, did not turn out to be significant predictors for VIMS, especially since the OKN *manipulation*, the visual fixation target, lead to a significant VIMS reduction. This apparent paradox can be explained if the fixation target has reduced the vection gain, which, in turn affected VIMS. The data show that VIMS did occur when the OKN was suppressed, whereas it did not at low levels of vection (compare Fig 6A and 6B). Notwithstanding, the OKN SPV still accounted for a small portion of the variance in the model. This would be in line with the findings of Ji et al [22] who showed that VIMS scores rose when vection was suppressed but OKN remained present. The ultimate test for involvement of OKN in VIMS would, however, require a comparison between this condition and one in which both OKN and vection are absent. Although we aimed at incorporating such a condition in the current study, we observed that our attempt to remove vection with the ambiguous motion stimulus did not entirely succeed. Vection was substantially reduced but not completely suppressed, and its effectiveness differed between participants. This contrasts with the findings of Ji and colleagues, who used a comparable method and report full vection suppression in their entire sample. One reason for this difference could be insufficient tuning of the relative size of the central FoV. We observed that many participants did not report vection during the final 1-minute training trial, but did so during the course of the 20 minute experimental trial. Using longer training trials could have prevented this issue, but as the tuning was done prior to the actual experimental session, we wanted to keep the pre-trial exposure to a minimum. More extensive tuning in a separate extra session would have been preferred, as was done by Ji et al.

The experimental setup and protocol were designed to minimize the occurrence of inadvertent head movements and slow, semi-static changes in head orientation over time. Accordingly, the recorded head movement data showed that inadvertent head movements occurred sparsely, had low amplitude and angular velocity, and that the drift in head orientation over time was minimal. Thus, it may not be surprising that both head movements and head tilt were no significant predictors for VIMS in the current study. Head movement frequency explained a small portion of the variance, but it is unclear whether these small head movements actually caused VIMS or were its consequence as participants started to feel uncomfortable. A much higher movement frequency, amplitude and velocity is usually required when head movements are incorporated in the experimental protocol to deliberately induce VIMS (e.g., [15]). Thus, we think it is unlikely that pseudo-Coriolis effects contributed to the VIMS scores in the current study. This means that, although successful in explaining other types of (visually induced) motion sickness (e.g., [51]), the SV theory does not provide an explanation for our observed results regarding Earth-vertical yaw vection Please note that it remains to be established to which extent our results generalize to visual stimuli that are not gravity-neutral, like visual rotation about an off-vertical axis. Here gravity comes into play, and leads to a more complex discrepancy between the visually induced motion sensation and what is actually sensed by the graviceptors. This alters both the perceived body motion [52] as well as the nauseogenity of the stimulus [29,53].

We also believe that there is no natural fit between the observed results and the postural instability theory. Support for this theory is provided by a range of studies showing that postural sway characteristics differ between participants who report motion sickness and those who do not (e.g.,[14,54–56]). In the majority of these studies, participants were either free-standing (e.g.,[14]), standing while being strapped to a vertical board with elastic bands (e.g., [55]), or sitting without back and/or head support (e.g., [54,56]), and they were exposed to visual stimuli like fore–aft linear oscillation (e.g.,[14,55]), roll motion (e.g., [54]), or combined motions occurring while playing a video game (e.g., [56]). These experimental conditions elicited postural sway. It is conceivable that postural responses might have been elicited by the visual yaw rotation as applied in the current study. However, our experimental setup was designed to minimize such potential responses by providing ample support of head and body in all experimental conditions. This makes it less likely that postural instability played a significant role in the current results. But since no direct postural sway measures were obtained in the current study, we can neither demonstrate nor rule out any contribution of postural instability in VIMS during visual yaw rotation.

### What is the provocative conflict?

With vection gain being the most important contributor to VIMS during visual yaw rotation, the SC theory may appear to be best suited for providing a theoretical framework for these results. Indeed, as proposed by this theory, vection seems to be a prerequisite for VIMS, as participants who suffered from VIMS also experienced vection. However, in our view, the SC theory does not provide a basis to explain our finding that higher levels of VIMS are related to higher levels of vection within participants. As argued in the introduction, the SC theory links VIMS to the discrepancy between visual and vestibular signals, and as such, it should not be the level of vection that is of main importance, but the occurrence of changes in vection. As a vestibular signal would only be expected during angular acceleration, the presumed discrepancy between visual and vestibular signals is absent during perceived constant velocity rotation. Nevertheless, the notion of “visual-vestibular conflict” is often put forward as the explanation for VIMS evoked by constant velocity visual yaw rotation (e.g., [7,9,10,57]). In our opinion, such a conflict is absent in that case. Proponents of SC theory are called upon to provide a hypothesis on how visual-vestibular conflict could come into play in displays of constant optic flow velocity.

The current results do not provide a clear-cut answer as to the cause of VIMS in such cases, and at this moment we can only speculate about the underlying mechanism. Here, we would like to suggest one line of inquiry that may prove fruitful in this respect. It concerns the “rest-frame” hypothesis proposed by Prothero and colleagues [19]. They propose that the reference frame that the central nervous system uses for spatial judgements, the so-called “rest-frame”, is defined by what is perceived as stationary. Difficulties in selecting a consistent stationary rest-frame would be a trigger for VIMS. As suggested by Prothero et al., VIMS “does not arise from conflicting motion signals per se, but rather from conflicting rest frames deduced from these motion signals” [19]. For example, such a rest frame problem might be present when part of the available visual information specifies self-motion, whereas other parts specify stationarity. This, for example, occurs when participants see the edges of the projection screen (or parts of the surrounding room) next to the inducing stimulus, and this indeed has been found to enhance VIMS [58]. It was suggested that the “intra-visual conflict” within the visual field (i.e., moving visual stimulus but stationary surroundings) is responsible for VIMS [58], but the results of the current study show that conflicting visual information per se (as in our ellipse manipulation) does not automatically lead to higher VIMS levels. The rest-frame hypothesis, in contrast, could explain the findings of the above mentioned study. Seeing the screen edges likely increases the uncertainty of whether the visual surround is stationary or not, and consequently, causes difficulty in selecting a consistent stationary rest-frame. In a similar manner, the uncertainty about what is stationary (i.e., the rest-frame) could be affected by top-down influences on VIMS, for example the knowledge or expectation about the likelihood of self-rotation. This is applicable to our experimental set-up, where participants were aware of the fact that they could not physically rotate. Thus, on the one hand, they experienced a compelling sense of self-motion, and on the other hand they knew it was in fact an illusion. Following this reasoning, a stronger or more compelling sense of vection would then cause a stronger conflict with their expectation, and thus, a higher level of VIMS. The general notion of a conflict between the actual experience and expectation is a key element in all motion sickness models ([11,26,51,59,60]), but the theories differ in what they regard to be its exact nature. If the actual perception and expectation about stationarity indeed matters, one would predict that VIMS would be affected by prior knowledge of the actual motion capabilities of the system. This remains to be investigated experimentally.

In conclusion, we found that vection gain (but not vection variability, eye or head movement) is the main contributor in VIMS during visual yaw rotation. Because of inter-individual differences in VIMS susceptibility, the relationship between VIMS and vection may stay hidden if assessed at a group level. To obtain a more profound understanding of why this relationship holds, a more detailed definition of the notion of “sensory conflict” is required.

## Supporting information

S1 Dataset Dataset containing all relevant individual data used for the analysis. A description of parameter names is provided on the second worksheet.
(XLSX)
